# Individual and Combined Effects of Medium- and Long-Chain Triacylglycerol and 2′-Fucosyllactose on Small Intestinal Morphology, Barrier Function, and Gut Microbiota in Growing C57BL/6 Mice

**DOI:** 10.3390/nu17172837

**Published:** 2025-08-31

**Authors:** Xinyuan Jin, Mengfan Shen, Mengdi Zhang, Haoqi Chen, Yufeng Jin, Yupeng Zeng, Zhijun Pan, Ziling Wang, Pan Wang, Yuting Yang, Zhiyuan Yan, Huilian Zhu, Dan Li

**Affiliations:** 1 Department of Nutrition, School of Public Health, Sun Yat-Sen University, Guangzhou 510080, China; jinxy8@mail2.sysu.edu.cn (X.J.); shenmf@mail2.sysu.edu.cn (M.S.); zhangmd36@mail2.sysu.edu.cn (M.Z.); chenhq55@mail2.sysu.edu.cn (H.C.); jinyf6@mail2.sysu.edu.cn (Y.J.); zengyp27@mail2.sysu.edu.cn (Y.Z.); panzhj5@mail2.sysu.edu.cn (Z.P.); wangzling@mail2.sysu.edu.cn (Z.W.); wangp328@mail2.sysu.edu.cn (P.W.); yangyt33@mail2.sysu.edu.cn (Y.Y.); 2 Guangdong Provincial Key Laboratory of Food, Nutrition and Health, Guangzhou 510080, China; 3 Inner Mongolia Mengniu Dairy (Group) Co., Ltd., Huhhot 011500, China

**Keywords:** medium- and long-chain triacylglycerol, 2′-fucosyllactose, gut microbiota, small intestinal morphology, intestinal barrier

## Abstract

**Background/Objectives:** Medium- and long-chain triacylglycerol (MLCT) and 2′-fucosyllactose (2′-FL) are functional ingredients abundant in human milk; however, their effects on small intestinal development and health remain largely unknown, and no research has explored their potential combined effects. **Methods:** In this study, growing C57BL/6 mice (3 weeks old) were fed diets without or with 2.5 g/100 g of MLCT, 2′-FL, or the combination (MLCT + 2′-FL; 5:1) for 21 days. Body weight, major organ indices, small intestinal morphology-related indicators (small intestinal length, villus height, crypt depth, villus height/crypt depth (V/C) ratio, and epithelial cell proliferation), and intestinal barrier function markers (goblet cell and Paneth cell count, protein expression of ZO-1 and occludin, and levels of sIgA and LPS) were measured. **Results:** In addition to the shared promotion of epithelial cell proliferation, MLCT intervention raised villus height and crypt depth, while 2′-FL intervention elevated Paneth cell count and sIgA levels. Notably, MLCT + 2′-FL intervention offered additional advantages (increasing the V/C ratio, goblet cell count, and expression of ZO-1 and occludin) without affecting crypt depth. 16S rRNA sequencing analysis of cecal contents revealed that all three interventions mainly affected beta diversity rather than alpha diversity, and enriched differentially abundant bacterial taxa: *Erysipelotrichaceae*, *Faecalibaculum*, *UBA1819*, and *Faecalitalea* in the MLCT group; *Enterobacteriaceae*, *Escherichia*, and *Allobaculum* in the 2′-FL group; *Bifidobacterium*, *Romboutsia*, *Clostridia*, and several other bacterial taxa in the MLCT + 2′-FL group. **Conclusions:** These results indicate that MLCT and 2′-FL interventions alone appear to provide different benefits for small intestinal development, and their combination may confer more comprehensive advantages.

## 1. Introduction

The early stage of life, especially the 1000 days from fetal life to 2 years of age, is considered a critical period for growth and development, with profound impacts on lifelong health [1]. As optimal nutrition for infants, human milk (HM) has been shown to promote infant growth and immune system development, modulate gut microbiota, and improve intestinal health through its nutrients and bioactive components [2,3,4]. Accordingly, the World Health Organization recommends exclusive breastfeeding for at least 6 months after birth and continued partial breastfeeding until 2 years of age or beyond. However, fewer than half of infants are exclusively breastfed, with many families switching to infant formulas (IFs) [5]. Thus, adding functional ingredients found in HM to formula milk, dietary supplements, and even foods for special dietary uses has become an effective and promising strategy, while it is limited by the research progress on components [6].

As the main site of nutrient absorption, the small intestine has an epithelium that forms protruding finger-like villi and invaginated crypts [7]. Villi expand the absorptive surface area of the small intestine, while crypt stem cells proliferate rapidly to support the growth and renewal of the intestinal epithelium [8,9]. The small intestinal barrier, which plays a core role in protecting the small intestine from harmful substances such as lipopolysaccharide (LPS), is a multi-layered structure consisting of the mucus layer, intestinal epithelium, and immune cells, among others [10,11]. The mucus layer covers the intestinal surface and contains antibacterial components like secretory immunoglobulin A (sIgA). Beneath it lies a monolayer of intestinal epithelial cells (IECs), interspersed with specialized cells such as goblet cells and Paneth cells that secrete various protective substances (e.g., mucin 2, lysozyme) [12,13]. These cells are linked by tight junction proteins, including zonula occludens-1 (ZO-1) and occludin, thereby maintaining intestinal integrity [14]. The gut microbiota is a vital biological element of the intestinal ecosystem, influencing intestinal development, differentiation, and barrier maturation through its bacterial composition, metabolites (e.g., short-chain fatty acids, SCFAs), and secreted proteins [15,16]. The immature absorptive and barrier functions of the small intestine, combined with high nutrient demands during the rapid growth period, make infants’ small intestines prone to overload [17,18]. In this situation, HM provides functional components and easily digestible nutrients, playing a vital role in promoting small intestinal development and health [19].

The lipids in HM make up 3–5% (*w*/*v*), and about 98–99% of them exist as triglycerides (TGs), namely human milk fat (HMF). HMF serves as the primary energy source for infants and has various biological roles [20,21]. It contains about 20–40% medium- and long-chain triglyceride (MLCT) and 7–23% medium-chain fatty acids (MCFAs), a unique feature compared to all other animal fats, milk, or vegetable oils [22,23]. Unlike common TG, which generally links fatty acids (FAs) of the same chain length, MLCT in HM simultaneously links MCFAs and long-chain fatty acids (LCFAs) and is typically composed mainly of 12:0, 14:0, 16:0, 18:1, and 18:2 FAs [22,24]. The majority of ingested MLCT are hydrolyzed into MCFAs, LCFAs, and monoglycerides in the small intestine [25]. After being absorbed by enterocytes, monoglycerides and LCFAs are re-esterified into TGs and packaged into chylomicrons for lymphatic transport. In contrast, MCFAs can be directly absorbed into the portal vein and undergo carnitine-independent mitochondrial transport, allowing for rapid energy delivery [26,27]. In this way, MLCT prevents the issue of insufficient essential fatty acids (EFAs) and the overly rapid release of MCFAs associated with medium-chain triglyceride (MCT), and reduces the digestive burden posed by long-chain triglyceride (LCT) [26]. Overall, these structural and compositional properties of MLCT better match infants’ physiological characteristics and nutritional needs, supporting growth and helping prevent diseases.

Previous studies have primarily focused on MLCT’s role in parenteral nutrition and its ability to inhibit visceral fat deposition, reduce obesity, and alleviate insulin resistance [28,29,30,31,32]. Recently, the high abundance of MLCT in HM has drawn increasing attention to its effects on infant and child development, yet research remains scarce [22,23,33,34,35]. Only one study has addressed the effects of MLCT on small intestinal development and health. This prior study, using 3-week-old mice fed a high-fat diet (HFD), reported that MLCT intervention modulates lipid metabolism and gut microbiota, and preliminarily detected the impact of MLCT on the villus and crypt, while no further investigations were conducted [33]. Many commercial IFs have an inappropriate proportion of MCFAs. Some IFs use physical blends to mimic HMF; however, these blends have different TG profiles from HMF. Plant-based IFs contain higher MCT content, while cow milk- or goat milk-based IFs have higher levels of short- and medium-chain TG [36]. Consequently, identifying the MLCT in HM, developing structured HMF substitutes, and understanding their functionality have become new challenges and opportunities in scientific research and industry related to IFs and functional foods or supplements [21,37].

Human milk oligosaccharides (HMOs), a group of indigestible carbohydrates, are the third most abundant component in HM, following lactose and lipids. They have been shown to regulate the gut microbiota, promote intestinal health, enhance immune function, and support neurocognitive development [38,39]. 2′-Fucosyllactose (2′-FL) is the most abundant and extensively studied HMO [40,41]. Previous studies using disease models (colitis, obesity, allergy, etc.) have found that 2′-FL can be metabolized by specific gut microbiota, elevating beneficial metabolites and modulating cell signaling pathways, thereby enhancing the intestinal barrier function [42,43,44,45,46]. However, limited studies have explored the role of 2′-FL in small intestinal development under normal physiological conditions. The focuses of these studies are dispersed across small intestinal morphology, barrier function, and gut microbiota, and the results are also inconsistent [47,48,49,50,51]. Therefore, comprehensive evaluations are needed.

Although previous studies have reported the combination of 2′-FL with other substances, they have primarily involved distinct oligosaccharides or probiotics [48,52,53,54,55], leaving many other types of compounds largely unexplored. However, the functional ingredients in HM naturally coexist and are typically consumed in combination rather than in isolation. Therefore, the present study aimed to investigate the individual and combined effects of MLCT and 2′-FL in growing C57BL/6 mice (from 3 to 6 weeks of age), with a focus on small intestinal morphology, barrier function, and gut microbiota.

## 2. Materials and Methods

### 2.1. Animals and Treatments

C57BL/6 mice (male, 3 weeks old) were obtained from Vital River Laboratory Animal Technology Co., Ltd. (Beijing, China) and raised in a specific pathogen-free facility (20–26 °C, 12 h day/night cycle) with free access to water and food. Mice were randomized into four groups of ten each: (1) the control (CON) group, fed a standard AIN93G diet (Jiangsu Medicience, Yangzhou, China); (2) the MLCT group, fed AIN93G with 2.5 g/100 g MLCT; (3) the 2′-FL group, fed AIN93G with 2.5 g/100 g 2′-FL; and (4) the combination of MLCT and 2′-FL (MLCT + 2′-FL) group, fed AIN93G with 2.5 g/100 g of the combination (MLCT:2′-FL = 5:1). In the AIN93G diet, soybean oil (7 g/100 g) is the sole lipid source. During the intervention, an equivalent amount of soybean oil was replaced with the MLCT supplement to maintain general consistency in macronutrient composition and energy content across groups. 2′-FL (99% purity) was obtained from DSM Vitamins Trading Co., Ltd. (Shanghai, China). MLCT-containing structured lipids were provided by Qingdao Seawit Life Science Co., Ltd. (Qingdao, China). The MLCT and MCFA (C8–C14) contents were 69.8% and 35.24%, respectively, whereas the LCT and MCT contents were 28.0% and 1.81%, respectively. The information on the FA composition of this MLCT supplement is provided in Supplementary Table S1.

The body weight (BW) and food intake of mice were recorded every 3 days. After 21 days of intervention, the mice were anesthetized for blood collection. Serum was isolated by centrifugation at 1000× *g* for 15 min and stored at −80 °C. Subsequently, the mice were euthanized, and a portion of the small intestine was immersed in 4% paraformaldehyde, embedded in paraffin, and sectioned at a thickness of 3 μm. The remaining small intestine tissues were dissected and preserved at −80 °C for further examination. Cecal contents were collected using sterile EP tubes, rapidly frozen, and preserved at −80 °C until utilization. The spleen, thymus, heart, liver, and brain were isolated and weighed.

### 2.2. H&E and AB-PAS Staining

Paraffin sections of the small intestine were immersed in a dewaxing solution and processed through a gradient ethanol series. Subsequently, the sections were respectively stained with H&E staining solution (G1076, Servicebio, Wuhan, China) and Alcian Blue-Periodic Acid-Schiff (AB-PAS) staining solution (G1049, Servicebio, Wuhan, China) following standard procedures. Afterward, the sections were mounted with neutral gum and coverslipped. Images were collected with the digital pathology section scanner (KF-PRO-120-H1, KF-bio, Ningbo, China). Quantitative analysis was performed using ImageJ software (version 1.8.0). For H&E staining, villus height was measured from the tip to the villus–crypt junction, and crypt depth was measured from the orifice to the base. At least 10 intact and well-organized villi and crypts from two sections per mouse were analyzed. For AB-PAS staining, the mean number of goblet cells per villus was determined based on at least 10 intact and well-organized villi from two sections per mouse.

### 2.3. Immunohistochemical (IHC) Staining

Small intestine sections were dewaxed and heated together with the antigen retrieval solution in a microwave oven. After blocking with 3% BSA at 37 °C for 30 min, the sections were incubated with primary antibodies against Ki-67 (1:200, Cell Signaling Technology, Danvers, MA, USA, 12202) and lysozyme (1:1000, Abcam, Cambridge, UK, ab108508) at 37 °C for 2 h or overnight at 4 °C, respectively. Further, they were treated with the horseradish peroxidase (HRP)-conjugated secondary antibody (1:1000, Abcam, ab6721), followed by staining and counterstaining using the diaminobenzidine (DAB) Chromogen Kit (ZLI-9017, ZSGB-Bio, Beijing, China) and hematoxylin. After being sealed with neutral gum and glass coverslips, the sections were photographed with the digital pathology section scanner (KF-PRO-120-H1, KF-bio, Ningbo, China). The average number of Ki-67-positive (Ki-67+) cells and Paneth cells (lysozyme-positive cells) per crypt was determined using ImageJ software (version 1.8.0), with 10 crypts from two sections per mouse analyzed.

### 2.4. Detection of LPS and SIgA

The levels of LPS in serum and sIgA in the small intestine were determined by enzyme-linked immunosorbent assay (ELISA) using Mouse LPS ELISA Kit (CSB-E13066m, Cusabio, Wuhan, China) and Mouse sIgA ELISA Kit (E-EL-M1040, Elabscience, Wuhan, China), respectively. The detections were carried out according to the manufacturer’s instructions, and the optical density of each well was measured using a TECAN SPARK 10M multimode microplate reader (Tecan, Männedorf, Zurich, Switzerland).

### 2.5. Western Blot

For protein expression analyses, small intestines were lysed with RIPA buffer (P0013B, Beyotime, Shanghai, China) to extract total protein. Protein concentration was quantified using the Pierce BCA Protein Assay Kit (23227, Thermo Fisher, San Jose, CA, USA). The protein samples were resolved by 7.5% or 10% SDS-PAGE gels and blotted onto PVDF membranes (IPVH00010, Millipore, Billerica, MA, USA). The membranes were blocked with 5% non-fat milk and then incubated overnight at 4 °C with various primary antibodies: anti-ZO-1 (1:1000, Abcam, ab96587), anti-occludin (1:1000, Abcam, ab216327), and anti-β-actin (1:5000, Cell Signaling Technology, 4970). This was followed by incubation with HRP-conjugated secondary antibodies (1:2000, Cell Signaling Technology, 7074) for 1 h. Next, protein bands were visualized with a chemiluminescence detection kit (Thermo Fisher, San Jose, CA, USA), and their density was analyzed with ImageJ software (version 1.8.0).

### 2.6. 16S rRNA Gene Sequencing

Cecal contents were used for DNA extraction and subsequent gut microbiota analysis. The purity and concentration of the extracted DNA were measured utilizing a NanoDrop One spectrophotometer (Thermo Fisher Scientific, MA, USA). The V3–V4 region of the bacterial 16S rRNA gene was amplified with specific primers (338F and 806R). Then, 1% agarose gel electrophoresis was used to detect the concentration and length of the PCR products. The Illumina platform (Guangdong Magigene Biotechnology Co., Ltd., Guangzhou, China) was used to sequence the target fragment libraries constructed according to the ALFA-SEQ DNA Library Prep Kit standard protocol. Sequencing data were filtered and merged through Fastp (version 0.14.1) and USEARCH-fastq_mergepairs (version 10) and then clustered into operational taxonomic units (OTUs) at 97% sequence similarity. Alpha diversity analysis was conducted using USEARCH-alpha_div (version 10) based on OTU abundance table. Nonmetric multidimensional scaling (NMDS) was applied for dimensionality reduction and visualization of beta diversity. The linear discriminant analysis (LDA) effect size (LEfSe) was performed for differential abundance analysis, with the LDA threshold set at 2.0.

### 2.7. Statistical Analysis

The data were represented as mean ± standard deviation (SD) and analyzed using R (version 4.4.2), with GraphPad Prism (version 9.2) used for charting. One-way ANOVA was performed, followed by Dunnett’s *t*-test to compare the differences between the CON group and each intervention group, and the Bonferroni test to evaluate the differences among the intervention groups. A *p*-value < 0.05 was considered statistically significant.

## 3. Results

### 3.1. General Indicators of Growing C57BL/6 Mice Following Interventions

**Figure 1:**
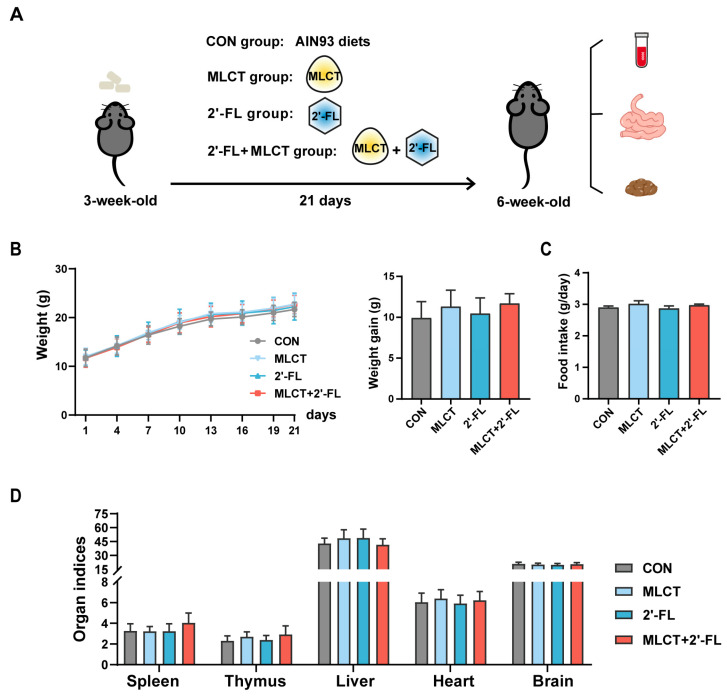
General indicators of growing C57BL/6 mice following interventions. (**A**) Experimental design. (**B**) Weight changes and weight gain during the intervention period. (**C**) Average food intake per mouse. (**D**) Organ indices, calculated as organ weight (mg) divided by BW (g). The data are shown as mean ± SD. *n* = 7–10. CON, control; MLCT, medium- and long-chain triacylglycerol; 2′-FL, 2′-fucosyllactose; MLCT + 2′-FL, the combination of MLCT and 2′-FL; BW, body weight.

Three-week-old C57BL/6 mice were fed a normal diet or treated with MLCT, 2′-FL, or MLCT + 2′-FL for 21 days (Figure 1A). No significant differences in BW or BW gain (9.92, 11.30, 10.46, and 11.69 g, respectively) were observed among the groups throughout the entire intervention stage (Figure 1B). The average daily food intake also showed no statistically significant differences among the groups, with each mouse consuming approximately 3.00 g/day on average during the intervention period (Figure 1C). Organ indices, including those of the spleen, thymus, liver, heart, and brain, did not differ significantly between the groups, although the MLCT + 2′-FL group showed an increasing trend in spleen index compared to the CON group (4.05 vs. 3.26, *p* = 0.055) (Figure 1D).

### 3.2. Small Intestinal Morphology and Epithelial Cell Proliferation in Growing C57BL/6 Mice Following Interventions

**Figure 2:**
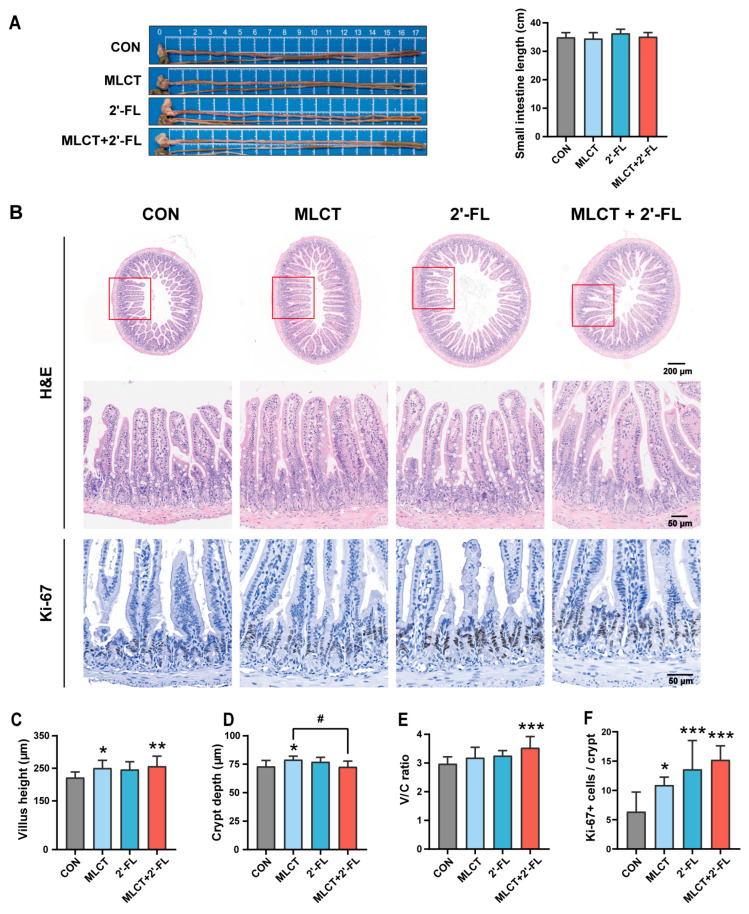
Small intestinal morphology and epithelial cell proliferation in growing C57BL/6 mice following interventions. (**A**) Representative images and length of the small intestine. (**B**) Representative micrographs of H&E staining and IHC staining for Ki-67 (scale bars, 200 μm and 50 μm). The red boxes indicate the corresponding magnified areas in the H&E-stained micrographs. (**C**–**E**) Villus height, crypt depth, and V/C ratio. (**F**) Number of Ki-67+ cells per crypt in the small intestine. The data are shown as mean ± SD. *n* = 7–10. * *p* < 0.05, ** *p* < 0.01, *** *p* < 0.001 (compared with the CON group). ^#^ *p* < 0.05 (between the combined group and the marked individual group). CON, control; MLCT, medium- and long-chain triacylglycerol; 2′-FL, 2′-fucosyllactose; MLCT + 2′-FL, the combination of MLCT and 2′-FL; V/C ratio, villus height/crypt depth ratio.

Among all groups, the average small intestinal length ranged from 34.48 cm to 36.32 cm, with no statistically significant differences observed (Figure 2A). H&E staining revealed that both the MLCT and MLCT + 2′-FL groups, but not the 2′-FL group, exhibited significantly higher villus height compared with the CON group (*p* < 0.05 and *p* < 0.01) (Figure 2B,C). In terms of crypt depth, the MLCT group showed greater depth than both the CON and the combined groups (*p* < 0.05), whereas no significant differences were observed between the other groups (Figure 2D). For the villus height/crypt depth (V/C) ratio, no appreciable difference was found, except that the MLCT + 2′-FL group exhibited an increased value compared to the CON group (*p* < 0.001) (Figure 2E). The proliferation level of IECs was assessed by the number of Ki-67+ cells. The average number of Ki-67+ cells per crypt was significantly higher in all intervention groups than in the CON group, but there was no difference between the intervention groups (Figure 2F).

### 3.3. Small Intestinal Barrier Function in Growing C57BL/6 Mice Following Interventions

**Figure 3:**
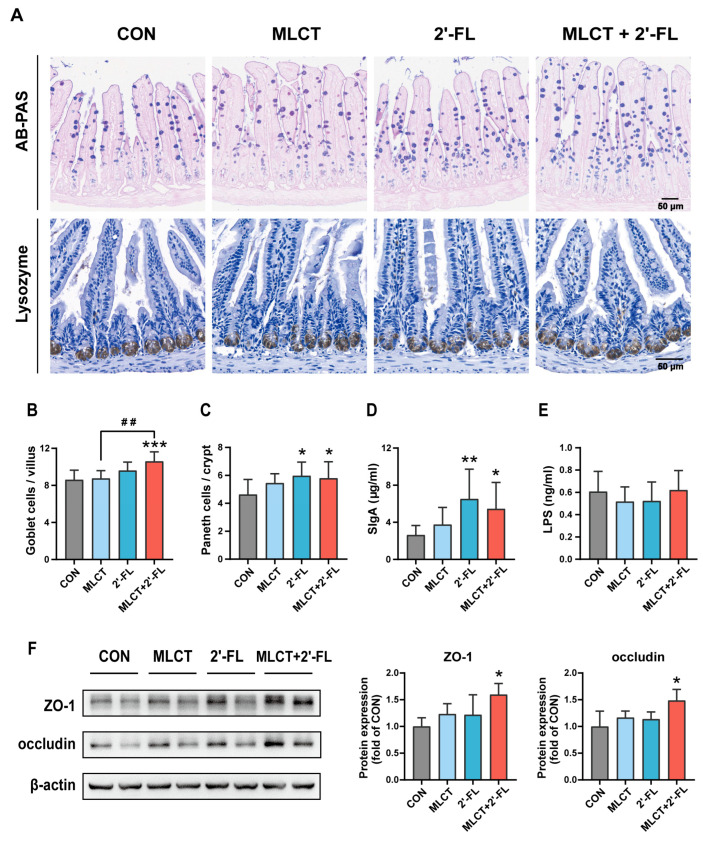
Small intestinal barrier function in growing C57BL/6 mice following interventions. (**A**) Representative micrographs of AB-PAS staining (goblet cells) and IHC staining for lysozyme (Paneth cells); scale bar, 50 μm. (**B**,**C**) The number of goblet cells per villus and Paneth cells per crypt. For these two cell types, at least 10 intact, well-organized villi and crypts from two sections per mouse were analyzed, respectively. (**D**,**E**) Levels of sIgA in the small intestine and LPS in serum, determined by ELISA. (**F**) Western blot detection and protein expression of ZO-1 and occludin in the small intestine. The data are shown as mean ± SD. For Western blot, *n* = 4. For other indicators, *n* = 7–10. * *p* < 0.05, ** *p* < 0.01, *** *p* < 0.001 (compared with the CON group). ^##^ *p* < 0.01 (between the combined group and the marked individual group). CON, control; MLCT, medium- and long-chain triacylglycerol; 2′-FL, 2′-fucosyllactose; MLCT + 2′-FL, the combination of MLCT and 2′-FL; sIgA, secretory immunoglobulin A; LPS, lipopolysaccharide; ZO-1, zonula occludens-1.

AB-PAS staining and IHC staining for lysozyme were used to determine the number of goblet cells and Paneth cells (Figure 3A). The MLCT + 2′-FL group showed a higher number of goblet cells per villus than both CON and MLCT groups (*p* < 0.001, *p* < 0.01), while no significant differences were observed among other groups (Figure 3B). The number of Paneth cells per crypt and sIgA levels were increased in the 2′-FL and MLCT + 2′-FL groups, but not in the MLCT group (Figure 3C,D). In each group of mice, no significant difference in LPS levels was observed (Figure 3E). Additionally, as detected by Western blot, the protein expression of ZO-1 and occludin was elevated only following the MLCT + 2′-FL intervention (Figure 3F).

### 3.4. Diversity and Composition of the Gut Microbiota in Growing C57BL/6 Mice Following Interventions

**Figure 4:**
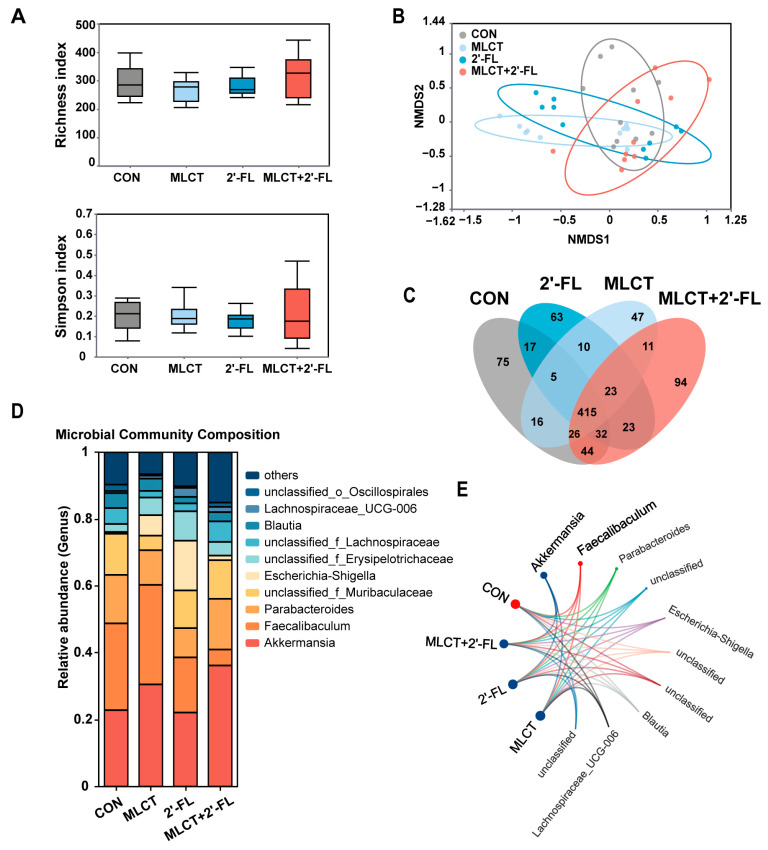
Diversity and composition of the gut microbiota in growing C57BL/6 mice following interventions. (**A**) Alpha diversity was assessed by the Richness and Simpson indices. (**B**) Visualization of beta diversity through NMDS. (**C**) Venn diagram of shared and unique OTUs. The overlapping areas represent the number of OTUs shared by groups, and the non-overlapping areas show the number of OTUs unique to each group. (**D**) Stacked histogram of microbial composition at the genus level. (**E**) Chord diagram of microbial composition. The node color of each group corresponds to the genus with the highest proportion within that group. *n* = 10. CON, control; MLCT, medium- and long-chain triacylglycerol; 2′-FL, 2′-fucosyllactose; MLCT + 2′-FL, the combination of MLCT and 2′-FL; NMDS, nonmetric multidimensional scaling; OTUs, operational taxonomic units.

Furthermore, we examined the impact of interventions on gut microbiota via 16S rRNA sequencing. There were no significant differences in alpha diversity as assessed by the Richness and Simpson indices (Figure 4A). For beta diversity, the NMDS plot revealed that the microbial communities in the four groups exhibited significant separation (Figure 4B). The OTUs also differed among the groups. As shown in the Venn diagram, 415 OTUs were shared by all groups, while 75, 47, 63, and 94 OTUs were unique to the CON group, MLCT group, 2′-FL group, and MLCT + 2′-FL group, respectively (Figure 4C). Regarding the microbial composition at the genus level, *Faecalibaculum* was the genus with the highest proportion in the CON group, while *Akkermansia* had the highest proportion in all intervention groups (Figure 4D,E).

### 3.5. Differentially Abundant Bacteria in Growing C57BL/6 Mice Following Interventions

**Figure 5:**
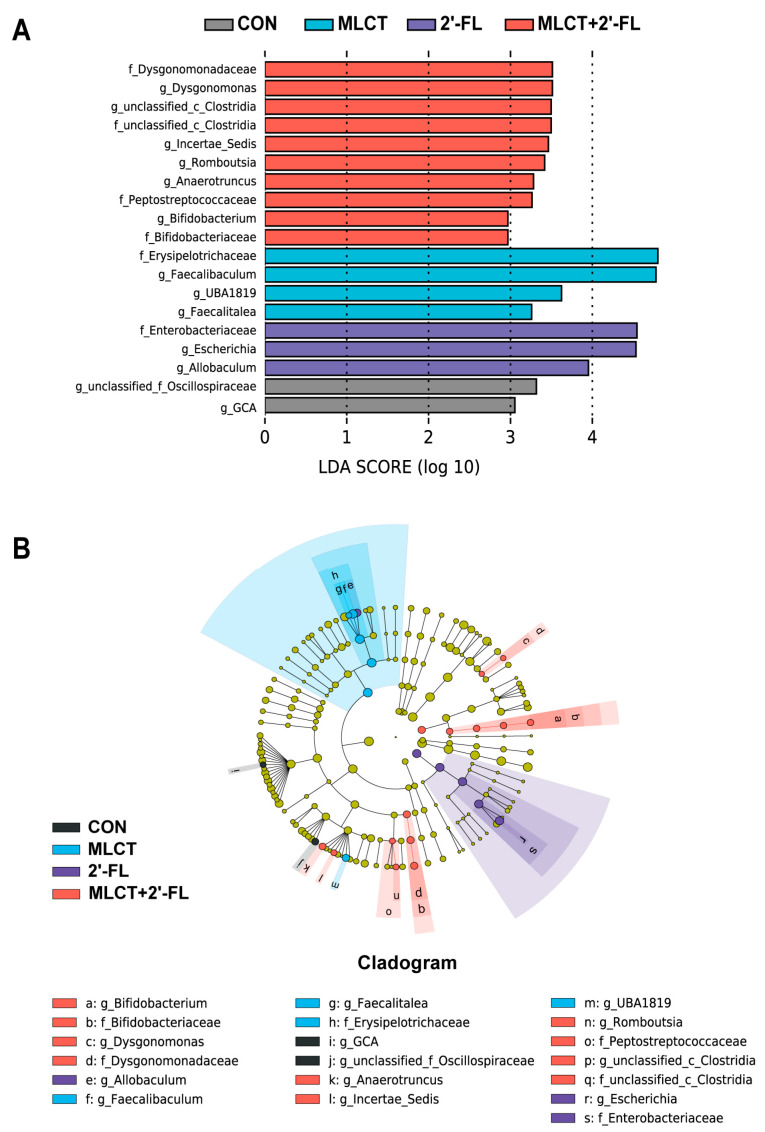
Differentially abundant bacteria in growing C57BL/6 mice following interventions. (**A**) The LDA score histogram generated by LEfSe (LDA > 2.0). (**B**) The cladogram based on LEfSe shows the taxonomic levels of differentially abundant bacteria. CON, control; MLCT, medium- and long-chain triacylglycerol; 2′-FL, 2′-fucosyllactose; MLCT + 2′-FL, the combination of MLCT and 2′-FL; LDA, linear discriminant analysis; LEfSe, linear discriminant analysis effect size.

LEfSe indicated that *Oscillospiraceae* and *GCA* were significantly more abundant in the CON group, whereas *Erysipelotrichaceae*, *Faecalibaculum*, *UBA1819*, and *Faecalitalea* were differentially enriched after the MLCT intervention (Figure 5A). Meanwhile, in the 2′-FL group, the relative abundances of *Enterobacteriaceae*, *Escherichia*, and *Allobaculum* were significantly higher. In mice treated with MLCT + 2′-FL, *Dysgonomonadaceae*, *Dysgonomonas*, *Clostridia*, *Incertae_Sedis*, *Romboutsia*, *Anaerotruncus*, *Peptostreptococcaceae*, *Bifidobacterium*, and *Bifidobacteriaceae* were prominently more abundant. The cladogram based on LEfSe is presented in Figure 5B, illustrating the taxonomic levels of differentially abundant bacteria, with levels decreasing from the inside to the outside (from phylum to genus). The heatmap showing the relative abundance of the top 20 genera is presented in Figure S1.

## 4. Discussion

MLCT and 2′-FL, as special ingredients in HM, have potential benefits for small intestinal development, but direct evidence is still lacking or inconsistent, especially regarding MLCT. In this study, growing C57BL/6 mice were fed MLCT and 2′-FL alone or in combination for 21 days. We found that the two compounds each exhibited their own unique advantages in promoting small intestinal development, and their combination provided more comprehensive benefits. Moreover, the three interventions enriched different gut bacteria, which may be associated with their functions.

The small intestine is an organ that absorbs the majority of nutrients. Villi and crypts serve as the key structural basis for its absorptive function [56]. Our study indicated that MLCT elevated villus height and crypt depth; MLCT + 2′-FL increased villus height and the V/C ratio; and 2′-FL alone did not affect any of these three indicators. All interventions enhanced IECs proliferation (Figure 2). To date, studies regarding the effects of MLCT on small intestinal morphology are still scarce. Only one study, conducted in HFD mice and focused on MLCT’s benefits for lipid metabolism, reported that MLCT intervention increased villus height and V/C ratio without affecting crypt depth, which partially supports our results [33]. While the benefits of 2′-FL in alleviating villus shortening and intestinal morphological damage have been studied in many disease models (intestinal mucositis, intestinal inflammation, etc.) [54,55,57,58], investigations into its effects on small intestinal development remain limited. A study in nursing rats showed that 2′-FL intervention increased the villus height and V/C ratio, but not crypt depth [47]. However, another study found opposite results in piglets [48]. Moreover, in young C57BL/6 mice, Liu et al. reported that the influence of 2′-FL on the villus and crypt appears to be dose-related [49]. Variations in animal models, 2′-FL dosage, and intervention methods might contribute to these differences. Villi are considered favorable for nutrient absorption, as they expand the contact area between the small intestine and the food [8,59]. Cells generated by epithelial cell proliferation in the crypts undergo maturation, differentiation, and migration from the crypts to the villi, thereby providing the cells necessary for growth and renewal [60,61]. Increased crypt depth may be linked to elevated proliferation levels [62,63]. However, if the rates of proliferation and maturation increase concurrently, crypt depth may remain unchanged [59,64]. Overall, although all three interventions promoted cell proliferation, MLCT and MLCT + 2′-FL, rather than 2′-FL, appeared to enhance small intestinal absorptive capacity through improved morphological development.

Goblet cells and Paneth cells, which produce key protective components, are the cornerstones of the small intestinal barrier [65,66]. SIgA is the main intestinal immunoglobulin that participates in immune responses and regulates the gut microbiota [67]. Meanwhile, tight junction proteins (ZO-1, occludin, etc.) interconnect IECs, forming a selective barrier that restricts the passage of harmful substances (e.g., LPS) [14]. Here, the above indicators were not affected by MLCT alone; however, Paneth cell count and sIgA levels were upregulated in both the 2′-FL and MLCT + 2′-FL groups. Notably, ZO-1 and occludin levels, as well as goblet cell count, increased only in the MLCT + 2′-FL group (Figure 3). Studies of MLCT are mainly focused on glucose and lipid metabolism, and no research has investigated the impact of MLCT on small intestinal barrier indicators, either in diseased or healthy models. Clinical studies have suggested that HMO supplementations are linked to a lower risk of infection [68,69]. Adding HMOs (with 2′-FL as the major component) to formula milk was found to increase fecal sIgA levels in infants, which supports our findings [70]. In animal experiments, the benefits of 2′-FL on goblet cell count, sIgA levels, and tight junction protein expression have been studied in various pathological models (e.g., HFD, colitis, and antibiotic-induced injury). These effects are linked to alterations in the gut microbiota and metabolites, regulation of the Toll-like receptor 4/nuclear factor-κB (TLR4/NF-κB) signaling pathway, and changes in cytokine levels [42,45,53,55,71,72,73,74]. However, limited research has explored its influence on the small intestinal barrier in growing animals. In young mice treated with 2′-FL, one study reported increased numbers of goblet cells and Paneth cells [49], and another found elevated goblet cell count and sIgA levels [50], both of which were similar to ours. Moreover, three studies reported inconsistent effects of 2′-FL intervention on mRNA levels of tight junction proteins in young mice and rats, and none assessed protein expression [47,49,51]. No studies have explored the effects of MLCT + 2′-FL intervention to date. However, HM treatment shows similarly comprehensive benefits in a pediatric intestinal organoid model, including promoting epithelial cell differentiation, increasing Paneth cell and goblet cell count, and strengthening tight junction functionality [75]. In summary, 2′-FL intervention rather than MLCT intervention improved the small intestinal barrier function, as manifested by an increased number of Paneth cells and elevated sIgA levels, whereas MLCT + 2′-FL intervention may offer more extensive benefits for this function.

The gut microbiota in early life is associated with the development and maturation of the intestine, immune system, and brain, serving as a long-term determinant of health and disease [15,76,77]. Our microbiota analysis found that all three interventions altered beta diversity but not alpha diversity (Figure 4A,B), suggesting their roles in promoting differences in microbial community composition rather than in species richness and evenness. At the genus level, *Akkermansia* accounted for the largest proportion in all three intervention groups (Figure 4D,E), which may partially explain the beneficial effects of the treatments. According to previous studies, *Akkermansia* has been shown to have various functions, such as promoting intestinal epithelial development, enhancing the intestinal barrier, and participating in metabolic regulation and immune system maturation through bacterial components or metabolites [78,79,80]. Similarly, enriched *Akkermansia* has been found in various models treated with 2′-FL, including models of colitis, food allergy, and aging [58,81,82].

Although the regulation of gut microbiota composition and diversity has long been a primary focus of research on HMOs, including 2′-FL, few published studies have reported the effects of MLCT, with the exception of three articles that used HFD mice [33,83,84]. In this study, the differentially abundant taxa, including the potentially beneficial genera *Faecalibaculum* and *Faecalitalea*, as well as *Erysipelotrichaceae* and *UBA1819*, were found to be enriched in the growing mice supplemented with MLCT alone (Figure 5). Specifically, *Faecalitalea* was associated with improved glucose metabolism [85]. *Faecalibaculum rodentium* has been reported to increase the number of Ki-67+ cells in vitro, which is consistent with the effects of MLCT observed in our study [86]. As for 2′-FL, its favorable effects on the gut microbiota have been observed in formula-fed infants, infants with milk protein allergy, and adults with chronic gastrointestinal disorders, as well as in young mice, rats, and piglets [48,50,51,87,88,89,90]. Among them, some studies have found the enrichment effect of 2′-FL on *Bifidobacterium* [51,87,88,89], an inconsistent result with ours. Interestingly, research has reported that *Bifidobacterium* was enriched by symbiotic interventions rather than by 2′-FL interventions, and the lower abundance of *Bifidobacterium* in animal pups (compared to infants) has been suggested as one possible reason [48,50]. Of note, our results revealed higher abundances of *Enterobacteriaceae*, *Escherichia*, and *Allobaculum* in the 2′-FL group (Figure 5). *Allobaculum* was linked to intestinal barrier protection and has anti-inflammatory properties [91,92,93]. The role of *Escherichia* varies depending on the specific bacterial species, including probiotic, commensal, and pathogenic types [94,95]. Moreover, we found that a series of unique taxa were more abundant in the MLCT + 2′-FL group, including *Dysgonomonadaceae*, *Dysgonomonas*, *Clostridia*, *Incertae_Sedis*, *Romboutsia*, *Anaerotruncus*, *Peptostreptococcaceae*, *Bifidobacterium*, and *Bifidobacteriaceae* (Figure 5). Of these, *Bifidobacteria* have been developed as probiotics, offering benefits for the digestive, immune, and neurological systems, with applications in infants, adults, and the elderly [96,97,98]. An in vivo study also found that supplementing with *Bifidobacterium longum* subsp. *infantis* can regulate the intestinal barrier and increase colonic IgA levels in growing mice [99]. Additionally, *Clostridia* were considered promising beneficial bacteria, while *Romboutsia* and *Anaerotruncus* have been observed to be associated with improved immune function [100,101,102].

We infer that the regulation of the gut microbiota may account for the different improvements in intestinal morphology and barrier function conferred by MLCT and 2′-FL, either alone or in combination. Microbiota-related metabolites could be potential mediators, with SCFAs representing key candidates. SCFAs are microbiota-fermented metabolites of HMOs (such as 2′-FL) and other indigestible dietary components [103]. They serve as energy sources for IECs, promote the expression of tight junction proteins and mucin 2, and regulate immune responses and antibody production [104,105]. Interestingly, MLCT intervention has also been found to increase fecal SCFAs levels in HFD mice [83]. In this study, several SCFA-producing bacteria (*Akkermansia*, *Faecalibaculum*, *Faecalitalea*, *Allobaculum*, *Clostridium*, *Romboutsia*, *Anaerotruncus*, and *Bifidobacterium*) were differentially enriched by different interventions, suggesting a potential mediating role of SCFAs [106,107,108,109].

The mechanism by which MLCT and/or 2′-FL modulate the gut microbiota may involve multiple aspects. Apart from acting as a prebiotic and being metabolized by specific bacteria, 2′-FL also promotes the growth of commensal bacteria through the cross-feeding mechanism and prevents pathogens from colonizing [38]. The fat absorption rate in adults is typically around 95%, whereas in infants, it is relatively lower, at approximately 85–90% [110,111]. Considering the disadvantages of non-MLCT for infants in digestion and absorption, they may have a lower absorption rate compared to MLCT. The unabsorbed non-MLCT and MLCT compounds, as well as their hydrolyzed products, may all have an impact on gut microbiota and interact with them [112]. Of note, the direct microbial regulatory and bactericidal effects of MCFAs have been reported [36]. In addition, compared to MCT and LCT, we hypothesize that the supplemented MLCT may elicit different bile secretion and endocrine responses, both of which can in turn influence the gut microbiota [24,36,113]. Whether the above events mediate our findings regarding the impact of MLCT and/or 2′-FL on the intestinal microbiota warrants further investigation.

Also, we speculate that mechanisms independent of microbe or its related metabolites may underlie the beneficial effects of MLCT and/or 2′-FL on intestinal histology. The high nutritional value of MLCT may play a crucial role in intestinal development. MLCT can serve as an important source of energy, biosynthetic materials, and bioactive lipids, along with their derivatives and metabolites [26]. Moreover, the role of MCFAs as signaling molecules for IECs has been demonstrated to support IECs proliferation and tissue repair [36,114,115]. Additionally, compared to LCT or a physical mixture of MCT and LCT, MLCT has been reported to promote the absorption of EFAs and lipid-soluble nutrients (such as vitamin D), although the mechanism has not been fully elucidated [116,117]. As for HMOs, their affinity for specific glycan-binding receptors may be involved. These receptors are expressed by various cells, including enterocytes and immune cells, and mediate gut immunity, such as the activation of Peyer’s patches and the stimulation of B cells to produce IgA [118,119]. In addition, HMOs can interact with Toll-like receptors (TLRs) to modulate both the development of the innate and adaptive immune systems and the immune balance in the infant gut. In particular, TLR4 may mediate a beneficial effect of HMOs on early-life immunity [38,120].

There are still limitations in our research: (1) Only male mice were studied. However, physiological processes, metabolic pathways, and responses to interventions may differ between males and females. Evaluating these sex differences is necessary to improve generalizability. (2) We employed only a single intervention dose and a fixed combination proportion. These precluded our ability to fully elucidate the effects of the interventions across different dose gradients and to determine the optimal proportion between the two components, which are critical for advancing both the effectiveness and practical application of the interventions. (3) Our study focused solely on small intestinal morphology, barrier function, and gut microbiota, while other aspects of small intestinal development, such as the immune system, were not addressed. (4) Relevant mechanism research remains insufficient and requires further exploration through the integration of multi-omics screening and metabolic studies of MLCT and 2′-FL, with a focus on key mediators and signals within or between specific cell types or microbial species. (5) Despite being recognized as a suitable model for intestinal development, mice have a short lifespan, resulting in a brief developmental period and growth characteristics that may differ from those in humans. Three- and six-week-old mice represent the average weaning transition age and the onset of puberty, respectively. Consequently, it is far from sufficient to extrapolate the findings to humans. Other animal experiments and clinical trials, as well as long-term interventions or interventions specific to the growth phase, remain necessary.

## 5. Conclusions

Our study suggests that the intervention of MLCT and 2′-FL alone or in combination provides different benefits for small intestinal development. Supplementation with MLCT or 2′-FL alone may enhance small intestinal morphology and barrier function, respectively. However, providing MLCT + 2′-FL may improve both aspects and offer unique benefits that are not observed with individual interventions. Differential gut bacteria enriched by the three interventions may be associated with their various benefits. These findings highlight the importance of MLCT and the HMO representative 2′-FL in promoting infant intestinal development and indicate more beneficial outcomes from a combined intervention. HM naturally exists as a mixture of multiple substances, which have been carefully refined through evolution [121]. Further exploration of HM components is needed to promote development and health during the early stages of life.

## Data Availability

The original contributions presented in this study are included in the article. Further inquiries can be directed to the corresponding authors.

## Supplementary Materials

The following supporting information can be downloaded at: https://www.mdpi.com/article/10.3390/nu17172837/s1, Table S1: The fatty acid composition of MLCT supplement; Figure S1: Heatmap of the relative abundance of the top 20 gut microbiota genera across all groups;

## Abbreviations

The following abbreviations are used in this manuscript:

2′-FL	2′-fucosyllactose
AB-PAS	Alcian Blue-Periodic Acid-Schiff
BW	body weight
CON	control
DAB	diaminobenzidine
EFAs	essential fatty acids
ELISA	enzyme-linked immunosorbent assay
FAs	fatty acids
HFD	high-fat diet
HM	human milk
HMF	human milk fat
HMOs	human milk oligosaccharides
HRP	horseradish peroxidase
IECs	intestinal epithelial cells
IFs	infant formulas
IHC	immunohistochemical
LCFAs	long-chain fatty acids
LCT	long-chain triglyceride
LDA	linear discriminant analysis
LEfSe	linear discriminant analysis effect size
LPS	lipopolysaccharide
MCFAs	medium-chain fatty acids
MCT	medium-chain triglyceride
MLCT	medium- and long-chain triacylglycerol
MLCT + 2′-FL	the combination of MLCT and 2′-FL
NMDS	nonmetric multidimensional scaling
OTUs	operational taxonomic units
SCFAs	short-chain fatty acids
SD	standard deviation
sIgA	secretory immunoglobulin A
TGs	triglycerides
TLRs	Toll-like receptors
V/C ratio	villus height/crypt depth ratio
ZO-1	zonula occludens-1
