# Supplementary material for: Individual and Combined Effects of Medium- and Long-Chain Triacylglycerol and 2′-Fucosyllactose on Small Intestinal Morphology, Barrier Function, and Gut Microbiota in Growing C57BL/6 Mice
Source: Nutrients. 2025 Aug 31;17(17):2837. doi: 10.3390/nu17172837 (PMC12430467; doi:10.3390/nu17172837)
Supplement: Supplementary file 1 [file nutrients-17-02837-s001.zip › nutrients-3808407-supplementary.pdf]

Table S1. The fatty acid composition of MLCT supplement.

Fatty acids	Relative content (%)
8:0	3.71
10:0	2.87
12:0	20.2
14:0	8.46
16:0	16.0
18:0	3.32
18:1	22.8
18:2	21.4
18:3	0.121

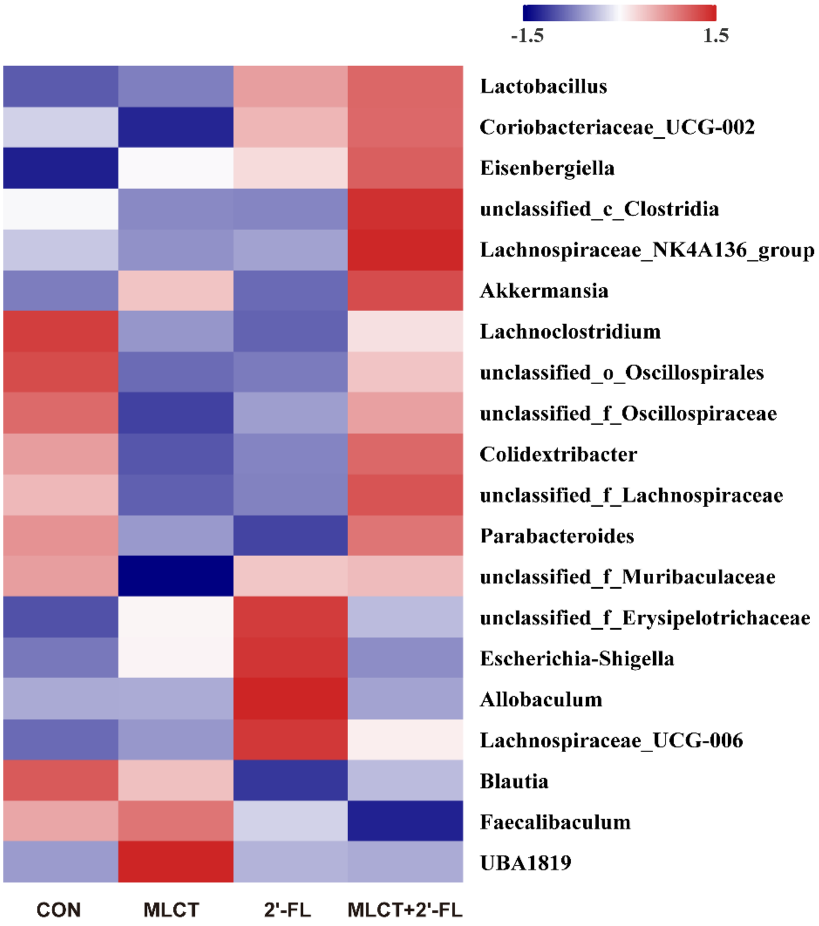

Figure S1. Heatmap of the relative abundance of the top 20 gut microbiota genera across all groups. The color intensity reflects the relative abundance, with red indicating higher abundance and blue indicating lower abundance.
